# Activation of *p21* by HDAC Inhibitors Requires Acetylation of H2A.Z

**DOI:** 10.1371/journal.pone.0054102

**Published:** 2013-01-18

**Authors:** Luca Bellucci, Mathieu Dalvai, Silvia Kocanova, Fatima Moutahir, Kerstin Bystricky

**Affiliations:** 1 Laboratoire de Biologie Moléculaire Eucaryote, Université de Toulouse, Toulouse, France; 2 LBME-UMR5099, CNRS, Toulouse, France; Peking University Health Science Center, China

## Abstract

Differential positioning of the histone variant H2A.Z in a p53 dependent manner was shown to regulate *p21* transcription. Whether H2A.Z is involved in *p21* activity in the absence of p53 is not known. The *p21* gene is repressed in estrogen receptor (ER) negative cell lines that are p53−/− and hormone independent for their growth. Here we demonstrate that class I and II pan Histone deacetylase inhibitors (HDACi) induce *p21* transcription and reduce cell proliferation of MDA-MB231, an ERα-negative mammary tumor cell line, in a H2A.Z dependent manner. H2A.Z is associated with the transcription start site (TSS) of the repressed *p21* gene. Depleting H2A.Z did not lead to transcription of *p21* but annihilated the stimulating effect of HDACi on this gene. Acetylation of H2A.Z but not of H3K9 at the *p21* promoter correlated with *p21* activation. We further show that HDACi treatment reduced the presence of the p400 chromatin remodeler at the *p21* TSS. We propose a model in which association of p400 negatively affects *p21* transcription by interfering with acetylation of H2A.Z.

## Introduction

Estrogen receptor negative breast cancer types are generally more aggressive and prone to metastasize. The absence of Estrogen receptor-alpha (ERα) correlates with hormone-independent growth of these mammary tumor cells and causes uncontrolled proliferation and insensitivity to anti-hormonal treatments. In ERα-negative cell lines, a subset of genes is epigenetically silenced [1], [2], while the majority of genes involved in cell cycle control and proliferation are constitutively expressed [1], [3], [4]. Aberrant gene expression is frequently the result of chromatin modifications and composition, including histone post-translational modifications and/or incorporation of histone variants [5]–[9]. In particular, deregulation of enzyme complexes responsible for histone acetylation and deacetylation can be associated with breast cancer progression and an increase in tumor malignancy [5]. Thus, compounds that change chromatin modifications are a promising anti-cancer approach. Histone deacetylase (HDAC) inhibitors, such as Trichostatin A (TSA), Suberoylanilide hydroxamic acid (SAHA), Panobinostat (LBH598) and sodium butyrate (NaB) can inhibit cancer cell growth in vitro and in vivo [10]–[12] as a result of selective induction of endogenous genes that play significant roles in G1-S progression [5]. One of the major regulators of cell cycle progression is the cyclin-dependent kinase inhibitor p21 CIP1/WAF1, a gene of the CIP/KIP family, which inhibits CDK activity. *p21* can be stimulated by p53 and its activity results in cell cycle arrest and/or apoptosis. Much of research on HDAC inhibitors has focused on the upregulation of *p21*. Activation of *p21* involves acetylation of promoter chromatin, but the mechanism remains poorly understood [13], [14].

The histone variant H2A.Z has been shown to bind to the promoter of *p21* at the p53 binding sites in p53+/+ cells (U2oS) [15]. In response to stress, H2A.Z is evicted to allow p53 to bind which leads to *p21* expression [15]. The p400 complex takes part in this pathway and was proposed to be responsible for H2A.Z deposition into the *p21* promoter. Depleting p400 by siRNA increases *p21* expression in a p53 dependent manner and induces premature senescence [16]. The mechanism of this activation is unclear.

In the ERα-negative breast cancer cell line MDA-MB231 p53 is mutated and non-functional. Here we show that activation of *p21* in response to HDACi treatment of these ERα-negative cells requires H2A.Z acetylation and exchange at its transcription start site.

## Materials and Methods

### Cell Lines, Transfection and Western Blotting

MDA-MB231, Hs-578T and HeLa cells were purchased from ATCC (used up to 15 passages). All cell lines were maintained in Dulbecco’s modified Eagle’s medium (DMEM) with Glutamax containing 50 mg/ml gentamicin, 1 mM sodium pyruvate and 10% heat-inactivated fetal calf serum (FCS) (Invitrogen). MDA-MB231 cells were treated with 50 or 100 ng/ml TSA (Sigma-Aldrich) and with LBH589 5×10^−8^ or 5×10^−9^ M for the indicated times. 4×10^6^ MDA-MB231 cells where mock-transfected (pcDNA3.1) or transfected with 2µg of pcDNA3.1/Tip60 (gifts from Dr. Didier Trouche) with Amaxa® Cell line Nucleofactor Kit V program X-013 according to the manufacturer’s protocol. MDA-MB231 cells were mock-transfected or transfected with H2A.Z siRNA ON-TARGET plus SMARTpool or scrambled (scr) siRNA (Dharmacon Thermo Scientific) with Interferine (Ozyme) according to the manufacturer’s protocol. Tip60 siRNA [17] was purchased from Eurogentec, and transfected with Interferine (Ozyme) according to the manufacturer’s protocol. For western blotting total cell extracts were isolated and proteins levels of p21 and GAPDH was analyzed by immunoblotting on gel SDS-page 15% with anti-p21 (SantaCruz Biotechnology, sc-397), anti-Tubulin (SantaCruz Biotechnology, sc-5286) and with anti-GAPDH (Millipore, mab374).

### RNA Analysis

Total RNA was extracted using an RNeasy mini-kit (Qiagen) and eluted with 35 µl of RNAase-free water. First strand cDNA was generated using 1 or 2 µg of total RNA in a reaction containing random oligonucleotides as primers with the ThermoScript RT-PCR system (Invitrogen). Real-time PCR was performed on a Mastercycler® ep *realplex* (Eppendorf) using the platinum SYBR Green q-PCR SuperMix (Invitrogen) according to the manufacturer’s instructions. Amplification conditions: 1 min at 50°C, 3 min at 95°C followed by 40 cycles (20 s at 95°C, 20 s at 60°C, 20 s at 72°C). q-PCR for *RPLP0* mRNA was used as an internal control. The primers used in q-PCR: *CDKN1A (p21, Cip1)*: 5′-GGAAGACCATGTGGACCTGT-3′ and 5′-GGATTAGGGCTTCCTCTTGG-3′, *H2AFZ*: 5′-CCTTTTCTCTGCCTTGCTTG-3′ and 5′-CGGTGAGGTACTCCAGGATG-3′, *RPLP0*: 5′-TGGCAGCATCTACAACCCTGAA-3′ and 5′- CACTGGCAACATTGCGGACA-3′, *TIP60:* 5′-CAGGACAGCTCTGATGGAATAC-3′ and 5′-AGAGGACAGGCAATGTGGTGAG-3′, *p400*: 5′-TGGCAGAGACTTGCTAAGGA-3′ and 5′-AGCGTCAACATCAGCTCACT-3′ [18].

### ChIP Assays

ChIP analyses were performed as described previously [19] from MDA-MB231 cells. Samples were sonicated to generate DNA fragments between 500 and 700 bp. Chromatin fragments were immunoprecipitated using antibodies against H2A.Z (ab4174, ABCAM), Acetyl H2A.Z (ab18262, ABCAM), RNA pol II (N20X, Santa Cruz), H3 (ab1791, ABCAM), Acetyl H3K9 (06–942, Millipore), TIP60 [20], p400 (ab70301, ABCAM), p300 (SC-584, SantaCruz Biotechnology) or an irrelevant HA antibody (H6908, Sigma) as control. The precipitated DNA was amplified by real-time PCR, with primer sets designed to amplify the promoter and the coding region of the *p21* gene. The primers used in q-PCR are listed here: *Primers 1:* 5′-TCAATGCCACCACCTTAACA-3′ and 5′-AGAGAGGCATCCTCCAGACA-3′, *Primers 2:* 5′-CTGTGGCTCTGATTGGCTTT-3′ and 5′-CTCCTACCATCCCCTTCCTC-3′, *Primers 3:* 5′-GAAATGCCTGAAAGCAGAGG-3′ and 5′-GTCTGCACCTTCGCTCCTAT-3′, *Primers 4 (TSS)*: 5′-ACTGGGGGAGGAGGGAAGT-3′ and 5′-AGCTGAGCCTGGCCGAGT-3′, *Primers 5:* 5′-CCAGGAAGGGCGAGGAAA-3′ and 5′-GGGACCGATCCTAGACGAACTT-3′, *Primers 6:* 5′-AGCCGGAGTGGAAGCAGA-3′ and 5′-AGTGATGAGTCAGTTTCCTGCAAG-3′, *Primers 7*: 5′-GCACCATCCTGGACTCAAGTAGT-3′ and 5′-CGGTTACTTGGGAGGCTGAA-3′.

### Proliferation and Cell Death Assays

MTT assay was performed using CellTiter 96® AQ_ueous_ One Solution Cell Proliferation Assay (Promega) according to the manufacturer’s instructions. For Trypan blue assay, 2×10^5^ cells seeded in 35 mm dishes. Cells were harvested and counted by trypan blue staining at indicated times following the different treatments.

## Results

### Histone Deacetylase Inhibitors Block Proliferation and Activate p21 Expression

**Figure 1:**
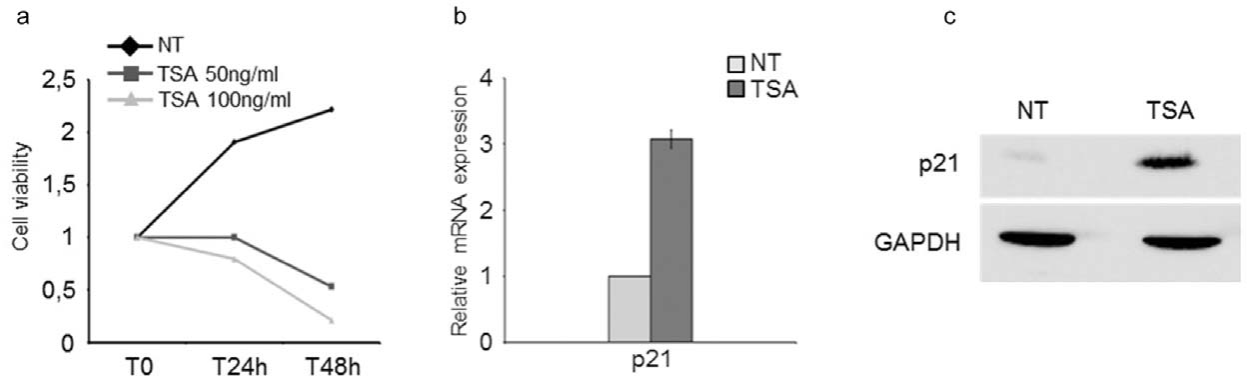
HDAC inhibitors reduce proliferation and activate *p21* transcription in ERα- negative/p53 mutated mammary tumor cells. a) MTT assay to quantify proliferation rates in the presence of Trichostatin A of MDA-MB231 cells. Two different concentrations (50 ng/ml and 100 ng/ml) were used. b-c) q-PCR and western blot analysis of *p21* mRNA expression and protein levels. Experiments were performed three times.

Proliferation of estrogen receptor alpha-negative (ERα-) breast cancer cell lines is estrogen-independent. A priority in breast cancer treatment is the development of agents able to contain or reduce growth of these cell types, which are more aggressive and insensitive to antiestrogens used to control estrogen responsive tumors. ERα- cells, MDA-MB231, were grown in standard medium and treated with the histone deacetylase inhibitors (HDACi) Trichostatin A (TSA) or panobinostat (LBH589). Cell growth was arrested by 50 or 100 ng/ml TSA (Fig. 1a) and 50 nM LBH589 (Fig. S1) during the first 48 h of treatment. At later time points, cell density decreased suggesting that prolonged exposure to HDACi induced cell death. Growth arrest and apoptosis are under the control of metabolic sensors, in particular the *p21* gene and its regulatory pathway. As previously described for pan HDACi [21]–[23] *p21* transcription was greatly activated in cells exposed to TSA for 24 h in which we detected >3-fold increase in *p21* mRNA levels (Fig. 1b) and a massive augmentation in p21 protein expression compared to untreated cells (Fig. 1c, S1d).

### Acetylated Histone H2A.Z Associates with the Transcription Start Site of p21

**Figure 2:**
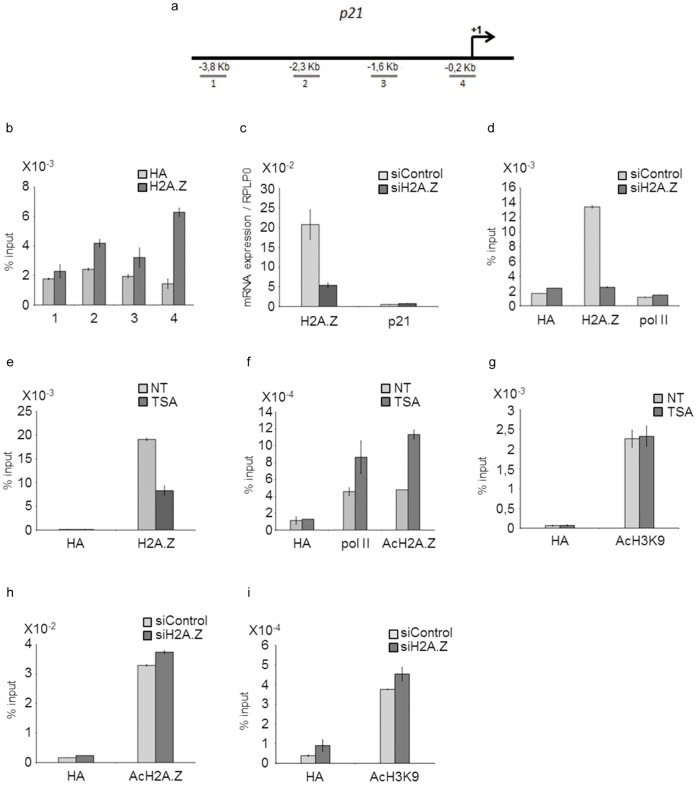
Acetylation of H2A.Z at the ***p21*** **promoter is necessary for its transcription.** a) Schematic representation of the *p21* promoter region showing PCR amplified fragments (1–4). b) Binding of H2A.Z to the *p21* promoter in MDA-MB231 cells. c) mRNA expression of H2AFZ (left) and *p21* (right) in MDA-MB231 cells transfected with a smartpool siH2A.Z for 72h. d) H2A.Z and polymerase II (pol II) binding to the *p21* TSS (fragment #4) in cells transfected with siH2A.Z or scramble siRNAs. e) H2A.Z binding to the *p21* TSS (fragment #4) in cells treated with TSA (50 ng/ml) for 48 h. f–g) acetylated H2A.Z, polymerase II (pol II) (f) and acetylated H3K9 (g) binding to the *p21* TSS (fragment #4) in cells treated with TSA. h-i) acetylated H2A.Z (h) and H3K9 (i) amount at *p21* TSS in cells transfected with siH2A.Z or scramble siRNA.

Transcription regulation of the *p21* gene is mediated by dynamic binding of the histone variant H2A.Z to p53 binding sites within the *p21* promoter region in p53 positive cells, such as ERα-positive MCF-7 (data not shown) or U2OS cells [15]. The ERα- MDA-MB231 cell line bears a mutated, non-functional p53 gene. We thus asked whether H2A.Z was also associated with the *p21* promoter in these cells as was shown to be the case in the p53- osteosarcoma, SaOS cell line [15]. Using chromatin immunoprecipitation (ChIP), we determined that H2A.Z was present at the transcription start site (TSS) of the *p21* gene. The amplified fragment (#4, Fig. 2) is adjacent to a set of six putative Sp1 binding sites that were shown to mediate *p21* transcription in a reporter assay [13]. The amount of H2A.Z detected at upstream sequences, including the p53 recognition elements (fragment #2), was significantly less abundant than at the TSS (Fig. 2b). According to the hypothesis that H2A.Z containing nucleosomes direct p53 binding [15], it was not surprising that H2A.Z was absent from these sites. As previously described in yeast [24], [25], decondensed chromatin is found at promoters of inactive but inducible genes. Based on this observation, we reduced the cellular pool of available H2A.Z by small interfering RNAs directed against H2A.Z and assessed *p21* mRNA expression (Fig. 2c). *p21* transcription remained insignificant (Fig. 2c). ChIP experiments further confirmed that despite a reduction in H2A.Z association with the TSS, polymerase II (pol II) was not recruited to this inactive gene (Fig 2d). Thus, *p21* transcriptional regulation does not only depend on the amount of H2A.Z associated with its TSS. Interestingly, treatment with 50 ng/ml TSA reduced H2A.Z binding to the activated *p21* (Fig. 2e, 1b). Release of H2A.Z was accompanied by recruitment of pol II and, strikingly, by an increase in the presence of acetylated H2A.Z at the *p21* TSS (Fig. 2f). This increase in acetylation was not seen for histone H3K9 (Fig. 2g). Moreover, the amount of acetylated H2A.Z and acetylated H3K9 present at the TSS did not vary in siH2A.Z transfected cells in which *p21* remained repressed (Fig. 2h–2i). We propose that acetylation of H2A.Z rather than its presence correlates with *p21* transcription activation in ERα- breast cancers. We postulated that the anti-proliferative effect of HDACi via *p21* expression depends on acetylation of H2A.Z. We thus asked whether this effect would be abolished in siH2A.Z treated cells.

### H2A.Z Controls HDACi Induced Growth Arrest via p21 Expression

**Figure 3:**
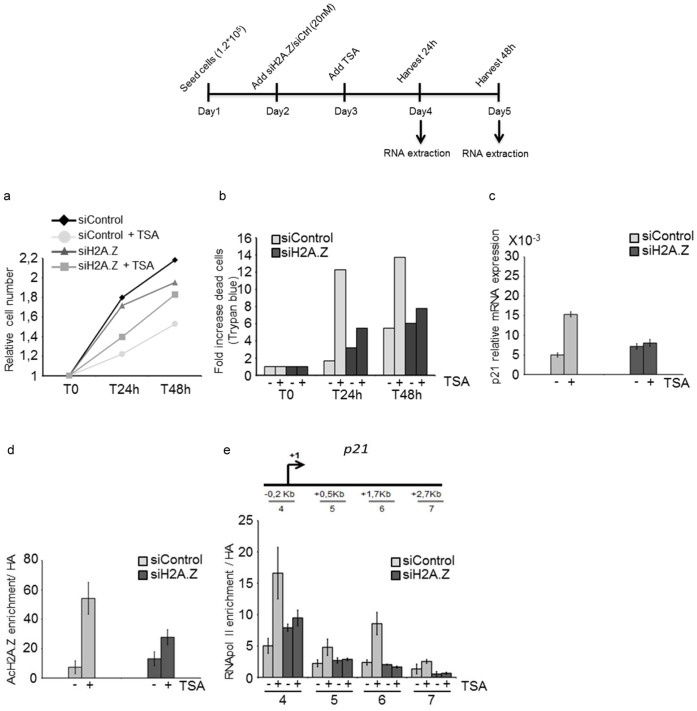
H2A.Z is required for the antiproliferative effect of HDACi. a) Cell growth assay b) Trypan blue assay c) qPCR analysis of *p21* mRNA expression. d) ChIP analysis of Acetyl-H2A.Z. e) ChIP analysis of RNA pol II. MDA-MB231cells were treated or not with TSA (50 ng/ml) for 24 h/48 h and/or transfected with a smartpool siH2A.Z (72 h) as indicated.

MDA-MB231 cells were grown in standard medium, treated or not with siH2A.Z for 24 hours before adding TSA or LBH. Surviving cells (MTT test, Fig. 3a) and dead cells (trypan blue, Fig. 3b) were counted before treatment as well as 24 h and 48 h following HDACi addition. Reduced cell growth in TSA treated cells was partly rescued in cells previously transfected with siH2A.Z (Fig. 3a). In particular, significant counts of dead cells were determined as soon as 24 h post treatment (Fig. 3b). While siH2A.Z transfected cells also showed a 2-fold increase in cell death compared to untreated cells, these cells were much less sensitive to TSA (Fig. 3b). Cell death at 48 h was almost similar in untreated cells compared to siH2A.Z transfected and TSA exposed cultures. Furthermore, *p21* mRNA levels did not vary in siH2A.Z transfected cells upon HDACi treatment compared to control cells treated only with TSA or LBH (Fig. 3c, S1c). H2A.Z is also required for *p21* activation upon TSA treatment in ER-negative, p53−/− Hs-598T cells (Fig. S2c). In contrast, knockdown of H2A.Z had no effect on *p21* activation in HeLa cells (Fig. S2a). Thus, H2A.Z specifically regulates *p21* in ERα-negative breast cancers following HDAC inhibitor treatment. We further found that acetylation of H2A.Z bound to the *p21* TSS was greatly reduced in siH2A.Z treated cells exposed to TSA (Fig. 3d). Pol II recruitment and elongation was abolished in TSA treated MDA-MB231 cells, from which H2A.Z was depleted (Fig. 3e). Thus, H2A.Z appears essential to mediate the anti-proliferative effect of HDACi by regulating *p21* expression. Notably, acetylation of H2A.Z was necessary for this regulation. We next wanted to gain insight into the mechanisms of H2A.Z acetylation at the *p21* promoter in ERα- cells.

### A Role for p400 but not Tip60 in p21 Transcription Regulation

**Figure 4:**
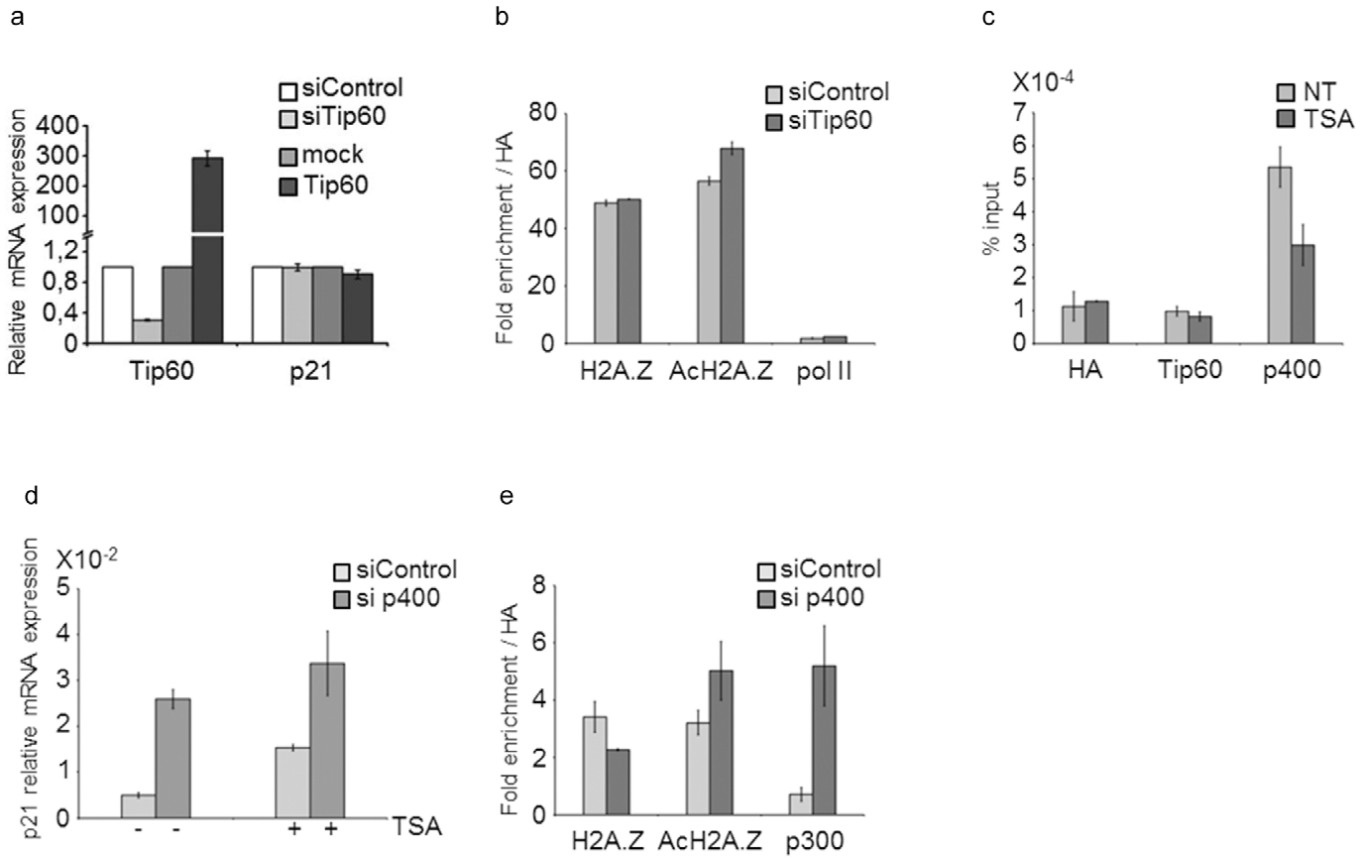
p400 but not Tip60 functions in *p21* expression in the absence of p53. a) Tip60 and *p21* mRNA expression. b) H2A.Z, Acetyl-H2A.Z and RNA pol II binding to the *p21* TSS in cell transfected with siTip60 or scramble siRNA. c) ChIP analysis of Tip60 and p400 recruitment to the *p21* TSS (fragment #4) in cells treated with TSA. d) *p21* mRNA expression in MDA-MB231 transfected with siRNA against p400 and treated or not with TSA. e) H2A.Z, Acetyl-H2A.Z and p300 enrichment at *p21* TSS (fragment #4) in MDA-MB231 transfected with si p400.

We first tested the impact of depleting or overexpressing Tip60, a histone acetyltransferase frequently found in complex with p400 and known to participate in H2A.Z mediated transcription regulation [15], [26]. Modulation of Tip60 mRNA levels did not alter *p21* expression levels, which remained almost undetectable (Fig. 4a). Accordingly, association of H2A.Z, acetylated H2A.Z and pol II did not vary at the *p21* TSS in Tip60 depleted cells (Fig. 4b). We next investigated which cofactor could be responsible for TSA induced activation of *p21*. Tip60 did not seem to be associated with the *p21* TSS in MDA-MB231 cells treated or not with TSA (Fig. 4c). In contrast, we detected significant amounts of the p400 remodeler at the *p21* TSS. Association of p400 decreased in TSA treated cells in which *p21* was activated (Fig. 4c). Reducing the available pool of p400 alone was able to activate *p21* expression (Fig. 4d) suggesting that the presence of p400 at the *p21* TSS represses this gene. Reduction of p400 allowed recruitment of the p300 acetyltransferase (Fig. 4e). Concomitantly, acetylation levels of H2A.Z markedly increased, due to eviction of a fraction of H2A.Z and an increase in acetylated H2A.Z at the *p21* TSS (Fig. 4e). We propose that TSA controls cell growth by modulating *p21* expression. *p21* activation requires release of p400 and H2A.Z, and an increase in acetylation of H2A.Z.

## Discussion

Lack of regulation of the *p21* gene whose expression is needed for cells to respond to insults by arresting proliferation is frequently observed in cancer. This loss is exacerbated by the absence of the functional tumor suppressor p53 protein in more aggressive tumor types. Histone acetyltransferase inhibitors have been shown to activate *p21* independently of p53. TSA (500 ng/ml) reduced growth of MG63 osteosarcoma, p53−/− cells and activated various *p21* promoter constructs driving a luciferase reporter gene [27]. We demonstrate that the endogenous *p21* gene is also activated by TSA or LBH589 in ERα- mammary tumor cells whose growth rate is insensitive to hormones and antihormones. In this study we provide a comprehensive analysis of the activation of the *p21* gene in ERα- MDA-MB231 cells which provides a mechanistic link between histone acetylation, H2A.Z variant incorporation and *p21* mediated growth arrest.

Unlike in p53 positive cells, U2OS osteosarcoma or MCF-7 cells, where the histone variant H2A.Z is associated with the p53 binding sites of the inactive *p21* gene, H2A.Z was present at the TSS in ERα- cells. H2A.Z binding to the TSS was frequently been observed in yeast [25] and human cells [28] where is thought to create a chromatin structure that is responsive to stimuli and co-factor binding. Its presence in promoter chromatin was also shown to be important for activation of ERα target genes such as *TFF1, PGR* or *CCND1* [29], [30]. Association of H2A.Z with promoter sequences could thus be related to an alternative pathway of gene activation in the absence of cognate transcription factors, including p53 or ERα. However, loss of H2A.Z binding was not sufficient to stimulate *p21* expression in MDA-MB231 cells. This observation correlates with previous studies showing that a 50% reduction H2A.Z association was not sufficient to induce changes in gene regulation [31].

In 2007 Gevry *et al.* showed that p400 and H2A.Z associate with the repressed *p21* gene in U2OS cells [15]. In response to DNA damage, p400 and H2A.Z were evicted to allow TIP60 recruitment and subsequent *p21* activation [15]. More recently Park *et al.*, however, demonstrated that p400 inhibits TIP60 activity by direct binding to TIP60 via its SANT domain [26].

In ER-negative, p53 mutant breast cancer cell lines, TSA treatment activates *p21*. Under these conditions, binding of p400 and H2A.Z to the *p21* promoter was reduced, but the concomitant increase in H2A.Z acetylation was independent of TIP60. Furthermore, depleting p400 also stimulated *p21* expression independently of TIP60 suggesting that p400 exerts a repressive effect. In contrast, depleting H2A.Z was not sufficient to mediate *p21* repression under normal conditions but it played a central role in *p21* activation upon TSA treatment. We propose that acetylation of H2A.Z rather than H2A.Z per se is important to drive proper *p21* gene expression. A ratio in favor of acetylated H2A.Z was associated with *p21* activation upon TSA treatment. This observation corroborates findings by Valdes-Mora and colleagues who recently correlated aberrant gene expression in prostate cancer cells with H2A.Z acetylation at specific promoters [32]. It is tempting to speculate that p400 favors *p21* activation by catalysing H2A.Z eviction and allowing the recruitment of HATs such as p300.

The histone-acetyltransferase CBP/p300 has been shown to act on the *p21* promoter at several Sp1 sites and independently of p53 as part of a multiprotein complex which also contains PR and Sp1 [33] in T47 mammary tumor cells. Here, in triple negative MDA-MB231 cells (ER-, PR-, HER-), p300 was also present at the *p21* promoter at levels proportional to transcriptional activity. Hence, the cofactors required for *p21* activation are distinct in p53 negative compared to positive cells [34]. These alternative pathways in cells in which regulation of *p21* does not obey to the classical pathways, open new avenues for growth control therapies.

Hua et al. [35] identified the histone variant H2A.Z as a potential epigenetic marker since its hormone-dependent expression correlates with increased probability of metastasis and decreased patient survival in a large scale study. For it to serve as a prognostic factor in breast cancer, the mechanisms unraveled by our study are relevant in ER- p53−/− cells that are otherwise difficult to act upon.

## Supporting Information

Figure S1 **p21 is activated in response to pan HDAC class I and II inhibitors.** a) MTT assay to quantify proliferation rates in the presence of LBH589 of MDA-MB231 cells cultured in rich medium. Two different concentrations (5*10−9 M and 5*10−8 M) were used. b) q-PCR analysis of *p21* mRNA expression levels. c) *p21* mRNA expression level in MDA-MB231 treated or not with LBH589 (5*10−9 M) for 48 h and/or transfected with a smartpool siH2A.Z (72 h) as indicated. d) Western blot analysis of p21 protein levels after 24 h of TSA treatment at the indicated doses.
(PDF)

Figure S2 **H2A.Z specifically regulates** ***p21*** **in ERα-negative breast cancers following HDAC inhibitor treatment.** a, b, c, d) q-PCR analysis of *p21* and H2A.Z mRNA expression in Hela (a, b) and in Hs-578T (c, d) cells. Cells were treated with siH2A.Z or scramble siRNA and treated for 24 h with two different concentrations of TSA.
(PDF)
