# Supplementary figures and images for: Activation of p21 by HDAC Inhibitors Requires Acetylation of H2A.Z
Source: PLoS One. 2013 Jan 18;8(1):e54102. doi: 10.1371/journal.pone.0054102 (PMC3548890; doi:10.1371/journal.pone.0054102)

Fig. S1

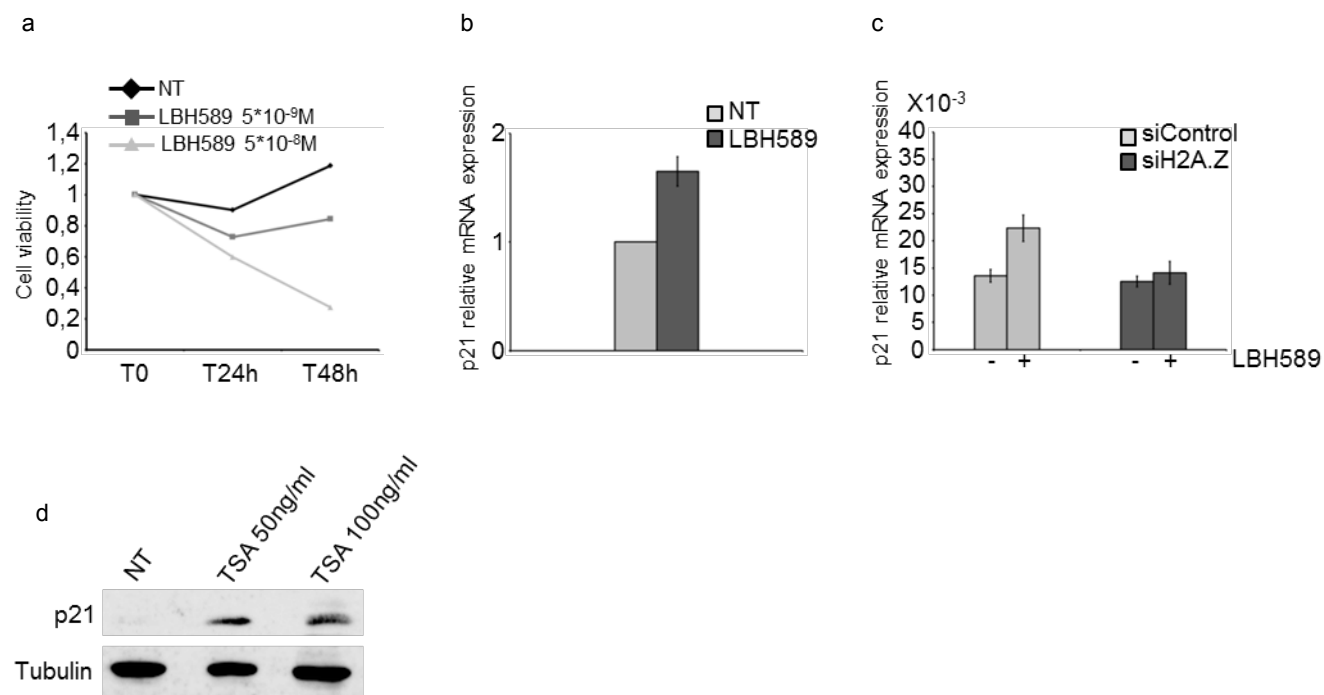

Supplement: Figure S1 — p21 is activated in response to pan HDAC class I and II inhibitors. a) MTT assay to quantify proliferation rates in the presence of LBH589 of MDA-MB231 cells cultured in rich medium. Two different concentrations (5*10−9 M and 5*10−8 M) were used. b) q-PCR analysis of p21 mRNA expression levels. c) p21 mRNA expression level in MDA-MB231 treated or not with LBH589 (5*10−9 M) for 48 h and/or transfected with a smartpool siH2A.Z (72 h) as indicated. d) Western blot analysis of p21 protein levels after 24 h of TSA treatment at the indicated doses. (PDF) [file pone.0054102.s001.pdf]

Fig. S2

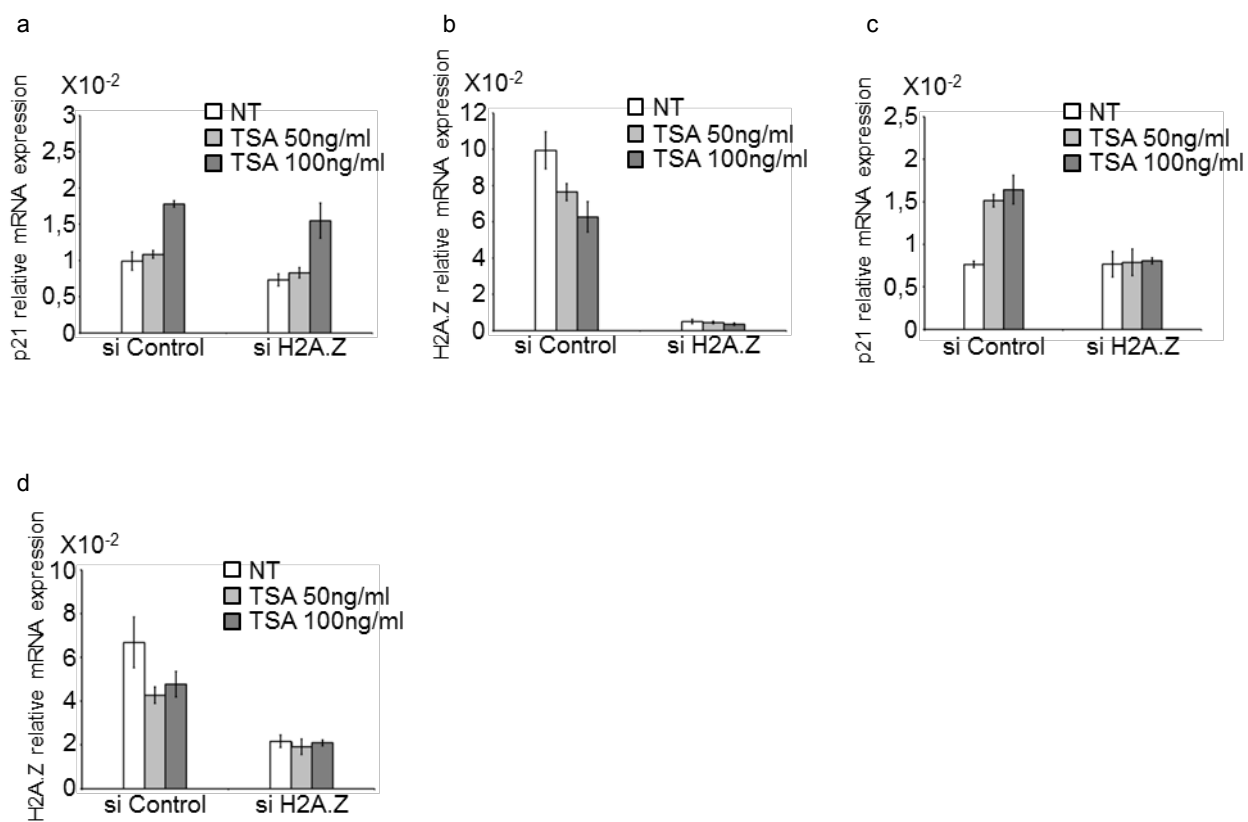

Supplement: Figure S2 — H2A.Z specifically regulates p21 in ERα-negative breast cancers following HDAC inhibitor treatment. a, b, c, d) q-PCR analysis of p21 and H2A.Z mRNA expression in Hela (a, b) and in Hs-578T (c, d) cells. Cells were treated with siH2A.Z or scramble siRNA and treated for 24 h with two different concentrations of TSA. (PDF) [file pone.0054102.s002.pdf]
